# Adenylylation of mycobacterial Glnk (PII) protein is induced by nitrogen limitation

**DOI:** 10.1016/j.tube.2012.12.003

**Published:** 2013-03

**Authors:** Kerstin J. Williams, Mark H. Bennett, Geraint R. Barton, Victoria A. Jenkins, Brian D. Robertson

**Affiliations:** a MRC Centre for Molecular Bacteriology and Infection, Imperial College London, Exhibition Road, South Kensington, London SW7 2AZ, UK; b Department of Life Sciences, Imperial College London, Exhibition Road, South Kensington, London SW7 2AZ, UK; c Centre for Integrative Systems Biology and Bioinformatics, Imperial College London, Exhibition Road, South Kensington, London SW7 2AZ, UK

**Keywords:** Nitrogen stress response, PII protein family, Post-translational modification, Mycobacteria

## Abstract

PII proteins are pivotal regulators of nitrogen metabolism in most prokaryotes, controlling the activities of many targets, including nitrogen assimilation enzymes, two component regulatory systems and ammonium transport proteins. *Escherichia coli* contains two PII-like proteins, PII (product of *glnB*) and GlnK, both of which are uridylylated under nitrogen limitation at a conserved Tyrosine-51 residue by GlnD (a uridylyl transferase). PII-uridylylation in *E. coli* controls glutamine synthetase (GS) adenylylation by GlnE and mediates the NtrB/C transcriptomic response. Mycobacteria contain only one PII protein (GlnK) which in environmental Actinomycetales is adenylylated by GlnD under nitrogen limitation. However in mycobacteria, neither the type of GlnK (PII) covalent modification nor its precise role under nitrogen limitation is known. In this study, we used LC-Tandem MS to analyse the modification state of mycobacterial GlnK (PII), and demonstrate that during nitrogen limitation GlnK from both non-pathogenic *Mycobacterium smegmatis* and pathogenic *Mycobacterium tuberculosis* is adenylylated at the Tyrosine-51 residue; we also show that GlnD is the adenylyl transferase enzyme responsible. Further analysis shows that in contrast to *E. coli*, GlnK (PII) adenylylation in *M. tuberculosis* does not regulate GS adenylylation, nor does it mediate the transcriptomic response to nitrogen limitation.

## 1 Introduction

All bacteria require a source of nitrogen for growth and have evolved several pathways to assimilate combined nitrogen such as ammonia. These pathways are regulated in response to the availability of nitrogen in the environment. Often, PII and PII-like proteins act as signal integrators regulating, through protein–protein interactions, nitrogen metabolism and assimilation in response to the perceived nitrogen status of the cell.^1–4^ PII proteins are 12–13 kDa homo-trimers with a highly conserved three-dimensional structure. The body of the PII trimer is compact and cylindrically shaped with three exposed loops (the T-loops), one per sub-unit.^5,6^ The T-loops are highly conserved in sequence but structurally very flexible. This conformational flexibility permits interaction with targets of many different structures. In addition to the T-loops, PII proteins possess three lateral inter-subunit clefts within which are two smaller loops (the B-loops and C-loops). The conformational state of PII is regulated by two mechanisms: the allosteric binding of the effector molecules, ATP, ADP and α-ketoglutarate (αKG), and the reversible covalent modification (uridylylation) of a conserved Tyrosine-51 residue in the T-loop.^6–8^ Crystal structure studies with *Escherichia coli* PII have shown that ATP or ADP binds to a nucleotide-binding pocket formed by the B- and T-loops from one sub-unit and a C-loop from another sub-unit. The binding site of αKG occurs close to the ATP binding site at the base of the T-loop, indicating how αKG binding may also alter T-loop conformation.^8^

*E. coli*, and many other bacteria, contains two PII-like proteins, PII (or GlnB) and GlnK, both of which are uridylylated under nitrogen limiting conditions at Tyrosine-51 by the uridylyl transferase GlnD.^1,9,10^ The Actinomycetales contain a single PII protein, GlnK. In *Corynebacterium glutamicum* and *Streptomyces coelicolor* grown under nitrogen limitation GlnK is adenylylated at Tyrosine-51, by GlnD, functioning as an adenylyl transferase.^11,12^ In the *Mycobacterium tuberculosis* H_37_Rv genome, Rv2919c is currently annotated as a probable nitrogen regulatory protein PII (GlnB) based on homology to *E. coli* (61.1% identity to GlnB and 54% identity to GlnK).^13^ However, the recent crystal structure determination of *M. tuberculosis* PII showed structural conservation with other GlnK proteins, and the location of Rv2919c in an operon with *amtB* (Rv2920) and *glnD* (Rv2918), means it is now generally accepted that the PII protein in *M. tuberculosis* is in fact a GlnK and not GlnB.^14^ Currently *M. tuberculosis* GlnD is annotated as an uridylyl transferase enzyme, but to date there is no evidence that uridylylation occurs as a post-translational modification mechanism in the Actinomycetales.

The binding of GlnK to ammonium transporters (AmtB and it homologues) is the most widely conserved function of PII proteins.^7,15^ The *amtB* gene is frequently co-transcribed with *glnK*, strongly suggesting that these proteins function together,^16^ and direct evidence for the interaction of GlnK and AmtB has been provided by crystal structure studies.^17,18^ Comparing the structure of *M. tuberculosis* GlnK (PII) (apo- and ATP-bound forms) with the *E. coli* GlnK:AmtB complex structure suggests that *M. tuberculosis* GlnK (PII) could form a complex with AmtB in a similar manner.^14^ These studies have also shown that the *M. tuberculosis* GlnK (PII) folds similarly to other PII family proteins^19^ and that ATP binding is vital for complex formation with AmtB.^14^

In nitrogen sufficient conditions, the un-modified T loop of GlnK inserts into the cytoplasmic exit channel of AmtB, blocking ammonium transport into the cell. Under nitrogen limitation, through the interplay of effector molecule binding and covalent modification of the Tyrosine-51 residue in the T-loop, GlnK dissociates from AmtB allowing ammonium influx through the channel.^7,12,17^ In *E. coli*, uridylylated GlnB (PII) and GlnK also control the transcriptional response to nitrogen availability, activating the NtrB/C two-component regulatory system that controls the expression of about 100 genes.^10,20^ However the Actinomycetales, including mycobacteria, do not contain an NtrB/C system. In *C. glutamicum*, adenylylated GlnK (PII) regulates the activity of the transcriptional repressor AmtR, which controls at least 33 genes.^21,22^ In *S. coelicolor*, approximately 50 nitrogen response genes are regulated through the activity of the transcriptional activator GlnR,^23^ however a direct role for GlnK (PII) in the regulation of this GlnR-mediated response has yet to be shown. Mycobacteria possess both GlnR (Rv0818) and AmtR (Rv3160) homologues but there has been very little study of the transcriptional response to nitrogen limitation in mycobacteria, and to date only a few genes have been identified to be under GlnR control, and the role of AmtR is unknown.^24,25^ The role of mycobacterial GlnK (PII) in the transcriptional response to nitrogen limitation has not been studied.

In most bacteria, two pathways assimilate ammonium; the glutamate dehydrogenase (GDH) and the glutamine synthetase/glutamine-2-oxoglutarate-amidotransferase (GS/GOGAT) pathways. In situations of nitrogen surplus the GDH pathway is favoured as the GS enzyme requires energy, estimated at approximately 15% of the total energy requirement of the cell.^10^ Therefore the expression and activity of GS are tightly controlled by feedback inhibition, transcriptional control and post-translation modification of GS by the bifunctional GS adenylyl transferase/adenylyl-removing enzyme GlnE.^26,27^ GS (Msmeg_4293) levels are induced in *Mycobacterium smegmatis* upon nitrogen limitation,^25,28^ but in *M. tuberculosis*, which does not possess a GDH pathway, GS (Rv2220) is constitutively expressed.^29^ The signalling cascade controlling GS expression and activity under nitrogen limitation are also unknown, but GlnE (Rv2221) is essential for growth in *M. tuberculosis* whereas it is dispensable in other bacteria.^26^ In *E. coli*, PII-UMP controls the amount of GS adenylylation through interaction with GlnE, and therefore controls GS activity. However in other Actinomycetes GlnE, and GS adenylylation, are not regulated by GlnK^11,12^; the role of mycobacterial GlnK in GS adenylylation under nitrogen limitation has not been studied.

The *M. tuberculosis glnK* gene is essential for growth in a primary murine macrophage model of infection.^30^ PII is absent from humans, and its putative central role in the regulation of nitrogen metabolism, makes it an attractive novel drug target in *M. tuberculosis*.^14^ However, little is known about either the covalent state of GlnK (PII) under nitrogen limitation or the function of GlnK (PII) in nitrogen sensing in mycobacteria. Here, we identify and analyse GlnK (PII) from *M. smegmatis* (Msmeg_2426) and *M. tuberculosis* (Rv2919), and determine the site at which it is covalently modified in a nitrogen-dependent manner. We also investigate the role of GlnD in the covalent modification of GlnK (PII) and the role of GlnK (PII) in the response to nitrogen limitation.

## 2 Material and methods

### 2.1 Bacterial strains and culture conditions

*M. smegmatis* mc^2^155 and *M. tuberculosis* H_37_Rv were grown at 37 °C with shaking at 180 rpm or 100 rpm, respectively, in modified Sauton's Minimal Medium (without asparagine and with ferric citrate replacing ferric ammonium citrate), and supplemented with 0.2% glycerol, 0.0001% ZnSO_4_ and 0.025% Tyloxapol.^31^ Nitrogen, in the form of ultra-pure ammonium sulphate (Sigma), was added at either 1 mM (nitrogen limiting) or 30 mM (nitrogen rich) final concentration. Cells were initially grown to late-log phase in nitrogen rich medium, the cells were washed twice with nitrogen-free medium and used to inoculate nitrogen limiting or nitrogen rich medium at an initial OD_600_ of 0.1. The *M. tuberculosis glnD* deletion strain used in the study was an unmarked, in frame deletion covering 1.7 kb of the *glnD* gene.^32^

### 2.2 Preparation of cell-free extracts

Samples were harvested after 12 h growth for *M. smegmatis* or 8 days for *M. tuberculosis* unless otherwise stated. Cells were harvested by centrifugation, re-suspended in 1 M urea and the cells lysed in a Fastprep ribolyser (Hybaid) for 2 × 30 s on setting 6. For *M. tuberculosis* samples, the protein extracts were filtered twice using 0.2 μm Spin X columns (Costar) for safe removal of the samples from the Biosafety Level 3 (BSL3) containment suite prior to mass spectroscopy (MS) analysis. Trypsin digestion using sequencing grade modified trypsin (Promega) was then performed on the mycobacterial protein extracts according to manufacturer's instructions, with incubation overnight at 37 °C. Formic acid (5% final concentration) was added to the sample and analysed by liquid chromatography (LC)-MS/MS as described below.

### 2.3 Determination of external ammonium concentrations

External ammonium levels in the growth medium were measured using an Aquaquant (AQ) ammonium detection kit (Merck) according to manufacturer's instructions. At each time point cells were harvested by centrifugation at 13K rpm and the cell supernatants removed for AQ analysis. For *M. tuberculosis* samples, the supernatants were filtered twice using 0.2 μm Spin X columns (Costar) to ensure safe removal of the samples from the BSL3 suite prior to AQ analysis. Ammonium concentrations in the cell supernatants were quantified by comparison to a standard curve of known ammonium sulphate concentration.

### 2.4 GlnK protein purification

*M. smegmatis glnK* (*Msmeg_2426*) was amplified from genomic DNA by PCR using the oligonucleotides: Forward 5′-GACTATCATATGATGGAATCTATGAAGC and Reverse 5′-GACTATAAGCTTCTACAGGGCGTCG and cloned into *Nde*I and *Hind*III sites (underlined) of pET28b. GlnK protein was over-expressed by transforming the plasmid into *E. coli* BL21 pLysS and inducing expression of GlnK at early log-phase (OD_600_ = 0.4) with 1 mM IPTG for 3 h at 30 °C. Cells were lysed using cell lysis solution (Sigma) and lysonase enzyme (Novagen) and purified His-GlnK protein was obtained using the MagneHis™ protein purification system (Promega) according to manufacturer's instructions. GlnK protein (200 ng) was digested overnight with sequencing grade trypsin and 5% (v/v) formic acid added to stop the reaction. Samples were analysed by LC-MS/MS as described below.

### 2.5 Snake venom phosphodiesterase (SVDE) treatment

Trypsin-digested protein extracts were incubated with 1 mg ml^−1^ snake venom in Tris buffer, pH9 and 1 mM MgCl_2_ for 3 h at 37 °C and analysed by LC-MS/MS as described below.

### 2.6 Mass spectrometry (LC-MS/MS)

Samples were analysed on an Applied Biosystems QTrap MS coupled to an Agilent 1100 LC stack. The Agilent stack consisted of a Binary pump, Capillary pump, Well Plate autosampler and a column oven with integrated 6 port valve. The system was configured to load samples onto a trap column (Agilent Zorbax SB 5 μm × 0.3 mm × 35 mm) using the binary pump; the trap column was washed and then switched into the capillary flow; peptides were separated on a capillary column (Agilent Zorbax SB 5 μm × 0.5 mm × 150 mm column). The LC was interfaced to the MS with a Turbo Ion Spray Source. The loading/washing solvent was H_2_O containing 0.2% Formic acid 0.02% TFA at a flow rate 150 μl min^−1^ and the resolving solvent was a gradient system of 0% B to 40% B over 45 min at a flow rate of 10 μl min^−1^ [(A) 94.9% H_2_O, 5% CH_3_CN, 0.1% formic acid; (B) 94.9% CH_3_CN, 5% H_2_O, 0.1% Formic acid]. The column oven was heated to 40 °C; the valve was switched to direct the flow from the trap into the resolving column after a 5 min wash. Typically the MS parameters were set to – Curtain Gas 10 psi, GS1 20 psi, GS2 20 psi, Interface heater on, TEM 150 °C, CAD 2, DP 65, EP 10, CXP 7, CE (collision energy) was set according to either empirically determined values or that estimated by MIDAS software (Applied Biosystems). The MS was used in “Trap” mode to acquire Enhanced Product ion scans for peptide sequencing and “Triple Quadruple” mode for MRM (Multiple Reaction Monitoring). Data analysis was performed using Analyst software (AB SCIEX).

### 2.7 RNA isolation

Three independent cultures of *M. tuberculosis* wild type and *glnD* deletion strains were grown under nitrogen limitation (1 mM) as described above for nine days after which time the nitrogen had completely run out, confirmed by Aquaquant. Briefly, cells were immediately added to 5 M GTC and harvested by centrifugation. Pellets were resuspended in TRIzol (Life Technologies) and stored at −80 °C. The thawed cell/trizol suspensions were transferred to tubes containing Zirconium beads (MP Biomedicals) and the cells lysed in a ribolyser Fastprep (Hybaid) for two cycles of 30 s on the maximum setting. Samples were then centrifuged and the TRIzol supernatants added to chloroform. After a second round of chloroform extraction, the RNA/DNA was then precipitated with isopropanol and the resulting nucleic acids resuspended in RNA secure (ABI Life Technologies). The RNA was then purified using the RNeasy kit (Qiagen) according to manufacturer's instructions except two rounds of DNAse treatment were performed: one on-column digest (*Invitrogen*) prior to elution of the RNA from the column and one Turbo DNase treatment (Ambion) on the eluted RNA. Superase (ABI Life Technologies) was added to the RNA to protect it from degradation and stored at −20 °C. RNA quality and quantity was determined by OD 260/280 and 260/230, gel electrophoresis and bio-analyser analysis.

### 2.8 Quantitative real-time PCR (qRT-PCR)

To determine gene expression levels, cDNA was amplified from 100 ng of RNA using the SuperScript III First-Strand Synthesis SuperMix (*Invitrogen*). qRT-PCR reactions were carried out in a final volume of 10 μl (1 μl of cDNA, 5 μl of TaqMan PCR master mix (Applied Biosystems), 0.5 μl of the appropriate TaqMan probe (Applied Biosystems)). Amplification was performed on an Applied Biosystems 7500 Real-Time System (conditions 50 °C 5 min, 95 °C 10 min, and 40 cycles of 95 °C 15 s, 60 °C 1 min). Linear amplification (*R*^2^ > 0.99) and similar amplification efficiencies for each TaqMan primer/probe set were determined. Real-time analysis was performed on RNA from three independent cultures and quantification of *sigA* expression served as an internal control. Fold changes were calculated as a ratio of the arbitrary expression units, standardised to *sigA*, between the nitrogen excess and limiting conditions using the delta delta Ct method.^33^ Primers and Taqman probe sequences for each gene studied are given in Table S3.

### 2.9 Preparation of labelled cDNA from total RNA

Labelled cDNA was prepared from 1.5 μg total RNA using Cy3-dCTP (GE Healthcare) and SuperScript II reverse transcriptase with random hexamer primers (Life Technologies – *Invitrogen* division). Agilent One Color Spike-In controls were labelled together with the RNA samples according to manufacturer's instructions. Labelled cDNA was purified by Qiagen MinElute column, combined with 10× CGH blocking agent and 2× Hi-RPM hybridisation buffer (Agilent) and heated at 95 °C for 5 min prior to loading onto microarray slides which were incubated overnight in an Agilent rotating oven at 65 °C, 20 rpm. After hybridisation, slides were washed for 5 min at room temperature with CGH Wash Buffer 1 (Agilent) and 1 min at 37 °C with CGH Wash buffer 2 (Agilent) and scanned immediately, using an Agilent High Resolution Microarray Scanner, at 2 μm resolution, 100% PMT. Scanned images were quantified using Feature Extraction software v 10.7.3.1.

### 2.10 Microarray design

The microarray was constructed by determining a pan-genomic redundant set of genes representing *M. tuberculosis* strains H_37_Rv (Ensembl Bacteria Release 2 (http://bacteria.ensembl.org/(EB))), CDC1551 (EB), H_37_Ra (EB), F11 (NC_009565; NCBI Entrez), KZN 1435 (NC_012943; NCBI Entrez), and *Mycobacterium bovis* strains AF2122/97 (EB), BCG (EB), BCG Tokyo 172 (NC_012207; NCBI Entrez). Briefly, all unique genes from the 4000 chromosomal predicted coding sequences of *M. tuberculosis* strain H_37_Rv were first selected; a subsequent iterative process was then used to determine genes absent from H_37_Rv or with significant divergence based on BLASTN bit scores. For each of the determined redundant gene set, multiple optimal hybridisation 60-mer oligonucleotide sequences were designed (Oxford Gene Technologies), from which a minimal non-redundant subset of oligonucleotides were selected with target coverage of three 60-mers per gene. Arrays were manufactured on the Inkjet in-situ synthesised platform (Agilent) using the 8 × 60 k format. The full array design is available in BμG@Sbase (BμG@Sbase: A-BUGS-41) and also in ArrayExpress (ArrayExpress: A-BUGS-41).

### 2.11 Statistical analyses of differential gene expression

Statistical analyses of the gene expression data was carried out using the statistical analysis software environment R together with packages available as part of the Bioconductor project (http://www.bioconductor.org). Data generated from the Agilent Feature Extraction software for each sample was imported into R. Replicate probes were mean summarised and quantile normalised using the preprocess Core R package. The limma R package^34^ was used to compute empirical Bayes moderated *t*-statistics to identify differentially expressed gene between time points. Generated *p*-values were corrected for multiple testing using the Benjamini and Hochberg False Discovery Rate. A corrected *p*-value cut-off of less than 0.01 was used to determine significant differential expression. Fully annotated microarray data have been deposited in BμG@Sbase (accession number E-BUGS-135; http://bugs.sgul.ac.uk/E-BUGS-135) and also ArrayExpress (accession number E-BUGS-135).

## 3 Results

### 3.1 Establishment of nitrogen limiting conditions

Our optimised nitrogen limiting conditions have been described^25^; mycobacteria were grown in Sauton's minimal medium with either 1 mM (nitrogen limiting) or 30 mM (nitrogen excess) ammonium sulphate added. At periodic intervals, hourly for *M. smegmatis* and daily for *M. tuberculosis*, bacterial growth was monitored by optical density measurements and the cell supernatants analysed for ammonium ions. We determined that ammonium had completely run out in the 1 mM medium (for nitrogen limiting growth) after 12 h for *M. smegmatis*^25^ and after 8 days for *M. tuberculosis* (Figure 3). Aquaquant analyses also confirmed that in the nitrogen excess medium ammonium was present in abundance at these time points. These time points were therefore selected for analyses unless otherwise stated.

### 3.2 Peptide selection for GlnK analysis

**Figure 1:**
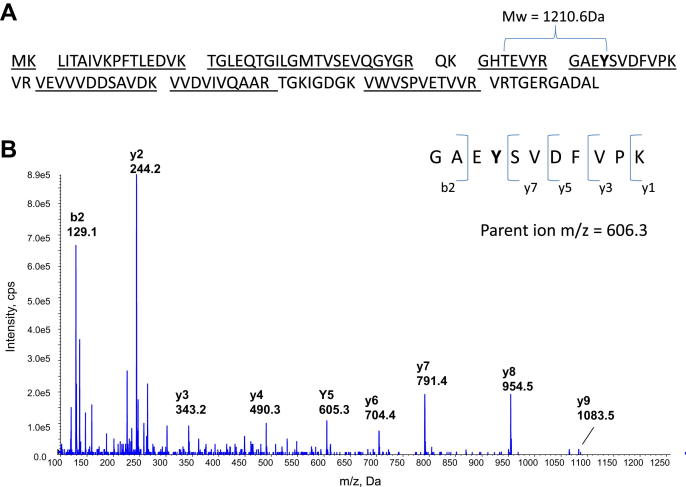
**(A)** Theoretical trypsin digest fragment of *M. smegmatis* GlnK shows the GAEYSVDFVPK Tyrosine-51 containing peptide with a mass of 1210.6 Da. **(B)** MS/MS analysis of the native GAEYSVDFVPK peptide obtained from *M. tuberculosis* grown in nitrogen excess.

Protein sequences for *M. smegmatis* GlnK (Msmeg_2426) and *M. tuberculosis* GlnK (Rv2919c) were obtained from the genome sequences in Smegmalist (http://mycobrowser.epfl.ch/smegmalist.html) and Tuberculist (http://tuberculist.epfl.ch/) respectively. *In silico* tryptic protein digests of these protein sequences (MIDAS software, Applied Biosystems) showed that the Tyrosine-51 (Y) residue, which is proposed to undergo modification in nitrogen limitation, was located in an identical peptide of 11 amino acids, GAEY*SVDFVPK with a predicted mass of 1210.6 Da, in both mycobacterial species (Figure 1A). To further assist peptide identification, the GlnK protein from *M. smegmatis* was over-expressed, purified and subjected to trypsin digest as described. LC-Tandem MS analysis of the tryptic digests of the purified GlnK protein confirmed correct identification of the native GAEYSVDFVPK peptide from *M. tuberculosis* grown in nitrogen excess (Figure 1B), which eluted with a retention time of 30.3 min.

### 3.3 Identification and characterisation of the GlnK peptide in mycobacterial cell extracts

**Figure 2:**
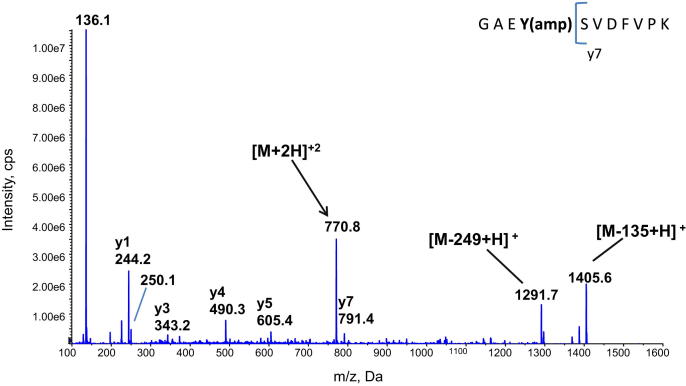
MS/MS analysis of the adenylylated GlnK peptide obtained from *M. tuberculosis* grown in nitrogen limitation.

LC-Tandem MS analysis was performed on cell extracts from *M. tuberculosis* grown in low and high ammonium in order to identify the GAEYSVDFVPK peptide. The native peptide was detected with a retention time of 30.3 min in cells grown in nitrogen rich medium, and a putative adenylylated peptide was detected with a retention time of 28 min in the cell extracts of *M. tuberculosis* grown under nitrogen limitation; this peptide peak was not evident in the nitrogen excess cell extracts (data not shown). MS analysis of the modified peptide (Figure 2) was consistent with adenylylation of the peptide on Tyrosine-51, represented by an increase in the *m*/*z* of the doubly charged parent ion by 164.5 Da, the loss of the y8 (954.5) and y9 (1083.5) ions, characteristic ions at *m*/*z* 136 and 250 and the appearance of two major new ions at 1405.6 and 1291.7 corresponding to the neutral loss of 135 and 249 typical of adenylylated peptides.^35^ Analysis with negative ionisation gave a strong product ion of 134, again indicative of adenylylation (Suppl. Figure S1). Targeted analysis to look for an uridylylated form did not identify any peptide showing this modification.

Multiple reaction monitoring (MRM) is a highly specific and sensitive mass spectrometry technique that can selectively identify and quantify compounds within complex mixtures.^36^ MRM allows the selection of specific peptides of interest based on peptide mass whilst all other peptides are filtered out. MRM was developed to detect and quantify both the non-adenylylated and adenylylated forms of the peptide using the transitions: 606.3 > 954.5, 770.8 > 136.1 respectively, additional qualifier ions 606.3 > 244.2, 606.3 > 791.4, for the native protein and, 770.8 > 244.2, 770.8 > 1291.7 for the adenylylated form were used. For further validation that the GAEYSVDFVPK peptide was adenylylated under nitrogen limitation, we exploited a well established method for the removal of the adenylyl groups using snake venom phosphodiesterase (SVPDE), which hydrolyses the phosphodiester bond between the tyrosine residue and the AMP group, releasing the adenylyl moiety.^37^ Figure 4 shows the MRM of *M. tuberculosis* GlnK grown in nitrogen limitation before (Figure 4A) and after (Figure 4B) treatment with SVPDE. Adenylylated GlnK (RT of 28 min) after SVPDE treatment was not detected suggesting efficient removal of the adenylyl moiety.

### 3.4 Adenylylation state of mycobacterial GlnK during nitrogen limitation

**Figure 3:**
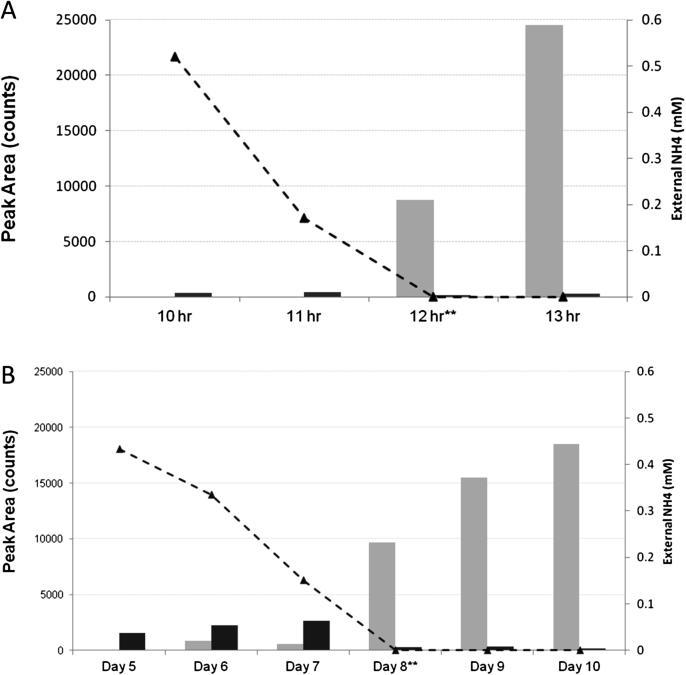
Dynamics of GlnK adenylylation in **(A)***M. smegmatis* and **(B)***M. tuberculosis* during nitrogen limitation. GlnK-AMP is represented by light grey bars and GlnK is represented by dark grey bars. Adenylylation of GlnK is induced upon external nitrogen exhaustion (**).

**Figure 4:**
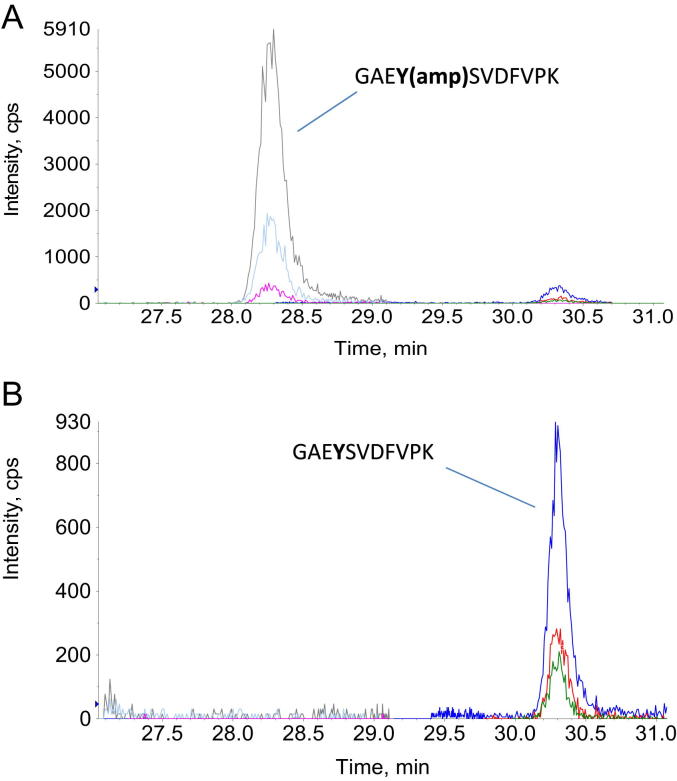
MRM of the GlnK peptide obtained from *M. tuberculosis* grown in nitrogen limitation. **(A)** Before SVPDE treatment **(B)** After SVPDE treatment. Different colour lines represent different peptide-specific transition pairs used in the analysis. (For interpretation of the references to colour in this figure legend, the reader is referred to the web version of this article.)

Using MRM, we then investigated the GlnK modification state in both *M. tuberculosis* and *M. smegmatis* when grown under nitrogen limitation. GlnK is thought to be key in the nitrogen signal transduction pathway, therefore adenylylation of GlnK should occur as nitrogen becomes limiting. Figure 3 shows the MRM results for the modified and non modified GlnK based on expected retention time for the respective peptides over nitrogen run out. Adenylylated GlnK is undetectable during initial growth of *M. smegmatis* and *M. tuberculosis*. As the external nitrogen levels deplete, very low levels of adenylylated GlnK are detected in *M. tuberculosis* (Figure 3B) but not in *M. smegmatis* (Figure 3A), however adenylylated GlnK levels were greatly increased in both species once the external ammonium had been completely exhausted. Low levels of non-adenylylated GlnK was detected in all samples (Figure 3). Adenylylated GlnK was not detected when mycobacteria were grown in nitrogen excess (data not shown).

### 3.5 GlnK is adenylylated in response to nitrogen limitation by GlnD

Since GlnK and GlnD are in an operon in the Actinomycetes, it has been assumed that GlnD acts as a nucleotidyl transferase to modify GlnK, or they work together, however the role of GlnD has not been experimentally determined in mycobacteria, although it is currently annotated as a putative uridylyl transferase, based on homology to *E. coli* GlnD. Having shown GlnK is adenylylated, we then investigated whether this was GlnD-dependent, using a *M. tuberculosis* GlnD deletion mutant (gift from Prof. Tanya Parish, Queen Mary, University of London). *M. tuberculosis* wild type and *glnD* deletion mutant were grown under nitrogen limiting conditions and cells harvested on day 8. The *glnD* deletion mutant has been previously characterised and shown to grow at the same rate as wild type in nitrogen limiting medium.^32^ However LC-Tandem MS showed that the *glnD* deletion mutant was unable to adenylylate GlnK, while it was adenylylated in the wild type strain (Suppl. Figure S2). Clearly, the lack of GlnK adenylylation does not lead to a major growth defect in nitrogen limitation and no alternative adenylylation system for GlnK was active under the conditions tested. The AQ analysis confirmed that both strains had completely exhausted the external ammonium by day 8, and samples taken 2 days later on still did not contain detectable adenylylated GlnK (data not shown).

### 3.6 GlnK adenylylation does not control GS adenylylation

**Figure 5:**
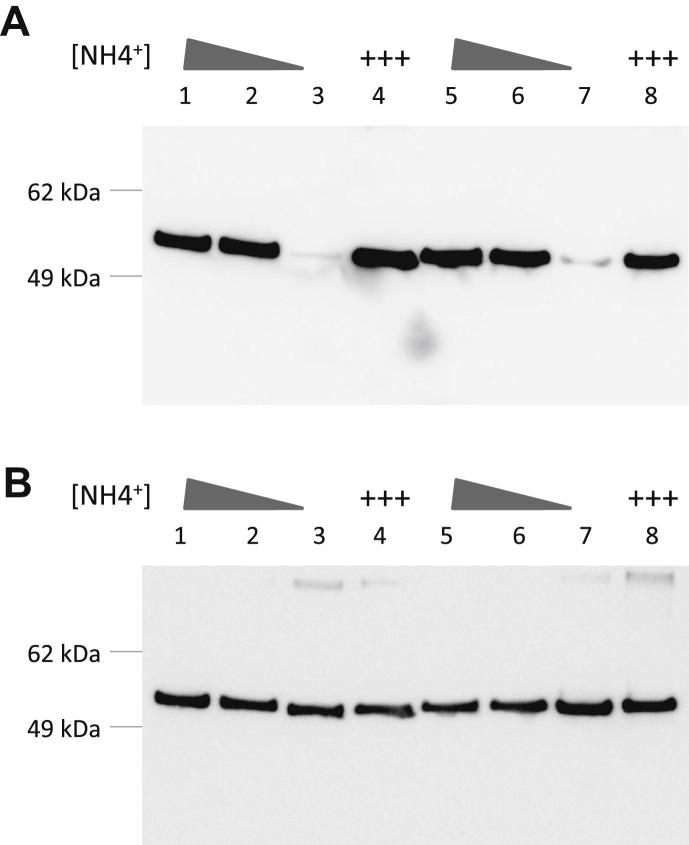
Western blot of GS adenylylation and GS protein levels in *M. tuberculosis* wild type (lanes 1–4) and *glnD* deletion mutant (lanes 5–8) during nitrogen limitation (representative gel; *n* = 3). **(A)** GS adenylylation levels significantly decreased upon external ammonium depletion (lanes 3 and 7) and increased upon replenishment of ammonium (+++) for both strains (lanes 4 and 8). **(B)** GS levels were not significantly changed during ammonium depletion or replenishment for either strain (lanes 1–8). Samples were collected on day 6 (lanes 1, 5), day 8 (lane 2, 6), day 9 before (lanes 3, 7), and after (lanes 4, 8) addition of ammonium.

In *E. coli* GS activity is tightly controlled by adenylylation of the enzyme by GlnE which is in turn controlled by protein–protein interaction with GlnK-UMP. Therefore in order to further investigate the role of GlnD and GlnK (PII) adenylylation, we studied GS adenylylation in mycobacteria during nitrogen limitation using a tyrosine-AMP specific antibody (gift from Dr. Christian Hedberg, Max-Planck Institute of Molecular Physiology). GS adenylylation was monitored in *M. tuberculosis* during ammonium depletion (days 6, 8 and 9) and upon replenishment of ammonium on day 9. Figure 5A shows that when external ammonium had completely run out (day 9), there was no GS adenylylation detected (lanes 3 and 7), but GS adenylylation was restored upon addition of 3 mM ammonium (+++) (lanes 4 and 8). Adenylylation levels in nitrogen excess conditions for both strains remained unchanged upon addition of 3 mM ammonium (data not shown). In order to confirm that the adenylylation pattern was not due to reduced GS protein levels or due to gel loading artefacts, the membranes were stripped and re-probed with the *M. tuberculosis* GS (*glnA1*) specific antibody (gift from Prof. Marcus Horwitz, UCLA). Figure 5B shows that the GS protein (53.4 kDa) levels were fairly constant during nitrogen limitation and replenishment. No detectable difference in either GS adenylylation (Figure 5A) or GS protein levels (Figure 5B) was observed between wild type and *glnD* deletion strains, suggesting that GS adenylylation is not modulated by GlnK (PII) adenylylation.

### 3.7 GlnK adenylylation does not regulate the transcription of nitrogen stress response genes

In order to determine if GlnK-AMP controls transcription of nitrogen stress response genes, through protein–protein interaction with the nitrogen response regulator GlnR or otherwise, we performed a microarray analysis to compare the expression of *M. tuberculosis* wild type and *glnD* deletion strains grown under nitrogen limitation. RNA samples (three independent biological replicates) were taken from nitrogen limited cultures on day 9 when the external ammonium was exhausted. We confirmed the induction of known nitrogen response genes (*amtB*, *nirB* and *glnK*) on day 9 in the wild type strain by qRT-PCR (Suppl. Figure S3) and re-confirmed that PII was adenylylated in the wild type strain only (data not shown). The microarray data was then analysed to determine which genes were significantly differentially expressed under nitrogen-stress comparing *glnD* mutant and wild type. Genes were considered to be significantly differentially expressed if their expression changed >2-fold with a *p*-value <0.01. Only 23 *M. tuberculosis* genes met these criteria; 9 genes were up regulated in the *glnD* mutant (Table S1) and 14 genes were down regulated (Table S2). However, none of the known nitrogen response genes or genes predicted to be involved in nitrogen metabolism,^38^ other than *glnD* itself, was identified. This shows that induction of the nitrogen stress response genes is independent of GlnK (PII) adenylylation. Comparative expression levels for all genes present on the microarray of the *M. tuberculosis glnD* deletion mutant and wild type strain under nitrogen limitation can be viewed in Supplementary Data File S4. Fully annotated microarray data have been deposited in BμG@Sbase (accession number E-BUGS-135; http://bugs.sgul.ac.uk/E-BUGS-135) and also ArrayExpress (accession number E-BUGS-135).

## 4 Discussion

### 4.1 Adenylylation of mycobacterial GlnK (PII) in nitrogen limitation

PII proteins form a large family of signal transduction proteins. They play a pivotal role in responding to the cellular nitrogen status in prokaryotes. However, despite their similarity at the protein sequence level, PII proteins of different bacteria play diverse roles in the response to nitrogen availability. In *E. coli*, both PII-like proteins undergo uridylylation under nitrogen limitation.^10^ Actinomycetales contain only one PII-like protein, which in *S. coelicolor* and *C. glutamicum* undergoes adenylylation under nitrogen limitation.^39^ However, in the Gram-positive *Bacillus subtilis*, GlnK (PII) does not appear to undergo any type of covalent modification under nitrogen limitation.^40^ Therefore, based on homology to the other Actinomycetales it was assumed that the mycobacterial GlnK (PII) would be adenylylated under nitrogen limitation, although this had not been tested experimentally. We have used LC-Tandem MS to identify the GlnK peptide predicted to undergo modification *in vivo*. We used MRM to demonstrate adenylylation of GlnK in both *M. smegmatis* and *M. tuberculosis* under nitrogen limitation but not under nitrogen rich conditions. We also confirm that the type of modification is adenylylation. The tryptic peptide (GAEYSVDFVPK) that undergoes modification is identical in both species of mycobacteria examined, and contains the highly conserved Tyrosine-51 residue in the T-loop, which we have also shown to be the residue that is modified. The kinetics of adenylylation differ between the two mycobacterial species studied here: in *M. smegmatis* there is no detectable adenylylation until external nitrogen is exhausted when there is a 3-log increase, whereas in *M. tuberculosis* low levels of adenylylation are detectable when nitrogen levels drop to 0.5 mM, two days before they become exhausted. Once nitrogen has run out there is 10-fold increase in adenylylation in *M. tuberculosis*. Both of these observations suggest that PII adenylylation is involved in the adaptation to nitrogen limitation. In *E. coli* the signal for uridylylation of PII is a combination of αKG levels, ATP to ADP ratios and intracellular glutamine levels.^41^ Our studies in *M. smegmatis* have shown increased intracellular αKG and decreased glutamine levels occurs upon nitrogen limitation,^42^ and therefore a similar signal for PII adenylylation may be present in mycobacteria, but this has not been verified experimentally.

### 4.2 The role of mycobacterial GlnK (PII) adenylylation in nitrogen limitation

The *glnK-amtB* operon is highly conserved amongst bacteria,^16^ but in the Actinomycetales this operon has an additional gene, *glnD*, which functions as an adenylyl transferase in *C. glutamicum* and *S. coelicolor*, but is annotated as a uridyl transferase in the mycobacterial genome.^13^ We show by LC-Tandem MS analysis of GlnK (PII) from the *M. tuberculosis glnD* deletion mutant that GlnD is the sole adenylyl transferase acting on GlnK under nitrogen limitation. Although there is another adenylyl transferase present in *M. tuberculosis*, GlnE, this enzyme does not compensate for the lack of GlnD as no adenylylation of GlnK (PII) is observed in the *glnD* deletion strain. We confirmed that the *M. tuberculosis* GlnD mutant grows at similar rates in nitrogen limiting conditions as reported,^32^ but now show that this growth is independent of GlnK adenylylation. This suggests that, contrary to *E. coli* and *C. glutamicum*, the adenylylation of GlnK does not play a major role in the adaptation of *M. tuberculosis* to nitrogen limitation; therefore its precise role remains unclear. In *S. coelicolor* a *glnD* deletion strain also showed no GlnK modification, and there was no growth defect under nitrogen limitation.^11^ This is in contrast to both *E. coli* and *C. glutamicum* where *glnD* deletion strains are impaired in their response to nitrogen.^22,43^

In *C. glutamicum* adenylylated GlnK has two functions: it dissociates from the ammonium transporter AmtB, permitting ammonium influx, and binds to the transcriptional repressor AmtR, relieving repression of at least 33 genes.^22^ Therefore, the adenylylation of GlnK by GlnD plays a central role in the *C. glutamicum* nitrogen response. In *E. coli*, adenylylated GlnB (PII) mediates the NtrB/C response of approximately 100 genes^10,20,44^ through interaction of PII-UMP with the response regulator NtrC. However, in *S. coelicolor* and mycobacteria, the major nitrogen transcriptional regulator protein is GlnR^24,25,45^ and this study shows that the GlnR-mediated transcriptional response in *M. tuberculosis* is independent of GlnK-AMP, as described for *S. coelicolor*.^11^ Only 23 genes were differentially expressed between the wild type and *glnD* mutant in nitrogen limitation and no genes thought to be involved in nitrogen metabolism or stress response were affected. Interestingly, the genes that were differentially expressed were involved in oxidative stress and siderophore production, suggesting that PII-AMP may play a more general role in protecting the cell against stress; this is currently under investigation.

Glutamine synthetase (GS) is an essential enzyme in the adaptation of bacteria to nitrogen limitation,^10,46^ catalysing the ATP-dependent condensation of glutamate and ammonia to yield glutamine. GS activity is tightly regulated by feedback inhibition, transcriptional control and post-translation modification by the bifunctional GS adenylyl transferase/adenyl-removing enzyme GlnE.^37,47^ In *E. coli*, activity of the GlnE enzyme is regulated through protein–protein interaction with GlnK-UMP.^48^ We now show that GS adenylylation is independent of GlnK (PII) adenylylation, and as reported previously,^49^ varied in response to nitrogen availability. Loss of GS adenylylation was not due to decreased GS protein levels, which were slightly increased upon nitrogen run out. Clearly mycobacteria, like *S. coelicolor*,^11^ use a novel mechanism to regulate nitrogen metabolism and in contrast to *E. coli*, GS adenylylation is not controlled by PII-AMP. Evidently GlnD and GlnK in the mycobacteria have functionalities distinct from those of their enteric counterparts, despite retaining post-translational modification and metabolite binding activities.

This study raises intriguing questions regarding the roles of GlnD and GlnK in mycobacteria. GlnD is thought to be the sensor of nitrogen sufficiency in *E. coli*, detecting glutamine levels, whereas PII is thought to be the sensor of nitrogen deficiency, detecting and binding increased levels of αKG. Therefore, GlnD could be activated by decreased glutamine levels in mycobacteria as there is a clear GlnD-dependent adenylylation of GlnK (PII) in nitrogen limitation; in *M. smegmatis* we have shown the αKG:glutamine ratios increase upon nitrogen limitation.^42^ However, whether there is a function for the adenylylated GlnK protein in nitrogen limitation is unknown. In all mycobacterial genomes *glnK* is co-transcribed with *amtB* suggesting they function together.^38^ Further evidence for this comes from crystal structure studies of *M. tuberculosis* PII which suggest that it could bind AmtB,^14^ but this has not been shown experimentally. However if one of the roles of GlnK (PII) adenylylation is to dissociate it from AmtB, permitting ammonium influx when nitrogen becomes limiting, then you would expect a phenotype for the *glnD* deletion strain in nitrogen limitation. Therefore, identifying GlnK protein–protein interactions in the cell could provide critical insights into the role of GlnD and GlnK in nitrogen metabolism in mycobacteria; experiments to address this are underway.

## Appendix A Supplementary data

Figure S1 Negative ionisation MS spectra of the GlnK peptide obtained from *M. smegmatis* grown in nitrogen limitation showing a strong product ion of 134 indicative of adenylylation.

Figure S2 MRM analysis of the GlnK peptide obtained from *M. tuberculosis* grown in nitrogen limitation **A.** Wild type strain **B.***glnD* deletion strain.

Figure S3 Confirmation by qRT-PCR of the induction of nitrogen response genes (**A**) *nirB* (**B**) *amtB* and (**C**) *glnK* in the wild type strain grown in nitrogen limitation for nine days. Data is a representative result of three experiments. Low (1 mM) ammonium black bars, High (30 mM) ammonium grey bars.

## References

[1] Arcondeguy T., Jack R., Merrick M. (2001). P(II) signal transduction proteins, pivotal players in microbial nitrogen control. Microbiol Mol Biol Rev.

[2] Ninfa A.J., Jiang P. (2005). PII signal transduction proteins: sensors of alpha-ketoglutarate that regulate nitrogen metabolism. Curr Opin Microbiol.

[3] Ninfa A.J., Atkinson M.R. (2000). PII signal transduction proteins. Trends Microbiol.

[4] Leigh J.A., Dodsworth J.A. (2007). Nitrogen regulation in bacteria and archaea. Annu Rev Microbiol.

[5] MacPherson K.H., Xu Y., Cheah E., Carr P.D., van Heeswijk W.C., Westerhoff H.V., Luque E., Vasudevan S.G., Ollis D.L. (1998). Crystallization and preliminary X-ray analysis of *Escherichia coli* GlnK. Acta Crystallogr D Biol Crystallogr.

[6] Xu Y., Cheah E., Carr P.D., van Heeswijk W.C., Westerhoff H.V., Vasudevan S.G., Ollis D.L. (1998). GlnK, a PII-homologue: structure reveals ATP binding site and indicates how the T-loops may be involved in molecular recognition. J Mol Biol.

[7] Forchhammer K. (2008). P(II) signal transducers: novel functional and structural insights. Trends Microbiol.

[8] Radchenko M., Merrick M. (2011). The role of effector molecules in signal transduction by PII proteins. Biochem Soc Trans.

[9] Atkinson M.R., Ninfa A.J. (1999). Characterization of the GlnK protein of *Escherichia coli*. Mol Microbiol.

[10] Reitzer L. (2003). Nitrogen assimilation and global regulation in *Escherichia coli*. Annu Rev Microbiol.

[11] Hesketh A., Fink D., Gust B., Rexer H.U., Scheel B., Chater K., Wohlleben W., Engels A. (2002). The GlnD and GlnK homologues of *Streptomyces coelicolor* A3(2) are functionally dissimilar to their nitrogen regulatory system counterparts from enteric bacteria. Mol Microbiol.

[12] Strosser J., Ludke A., Schaffer S., Kramer R., Burkovski A. (2004). Regulation of GlnK activity: modification, membrane sequestration and proteolysis as regulatory principles in the network of nitrogen control in *Corynebacterium glutamicum*. Mol Microbiol.

[13] Cole S.T., Brosch R., Parkhill J., Garnier T., Churcher C., Harris D., Gordon S.V., Eiglmeier K., Gas S., Barry C.E., Tekaia F., Badcock K., Basham D., Brown D., Chillingworth T., Connor R., Davies R., Devlin K., Feltwell T., Gentles S., Hamlin N., Holroyd S., Hornsby T., Jagels K., Krogh A., McLean J., Moule S., Murphy L., Oliver K., Osborne J., Quail M.A., Rajandream M.A., Rogers J., Rutter S., Seeger K., Skelton J., Squares R., Squares S., Sulston J.E., Taylor K., Whitehead S., Barrell B.G. (1998). Deciphering the biology of *Mycobacterium tuberculosis* from the complete genome sequence. Nature.

[14] Shetty N.D., Reddy M.C., Palaninathan S.K., Owen J.L., Sacchettini J.C. (2010). Crystal structures of the apo and ATP bound *Mycobacterium tuberculosis* nitrogen regulatory PII protein. Protein Sci.

[15] Javelle A., Merrick M. (2005). Complex formation between AmtB and GlnK: an ancestral role in prokaryotic nitrogen control. Biochem Soc Trans.

[16] Thomas G., Coutts G., Merrick M. (2000). The *glnKamtB* operon. A conserved gene pair in prokaryotes. Trends Genet.

[17] Conroy M.J., Durand A., Lupo D., Li X.D., Bullough P.A., Winkler F.K., Merrick M. (2007). The crystal structure of the *Escherichia coli* AmtB-GlnK complex reveals how GlnK regulates the ammonia channel. Proc Natl Acad Sci U S A.

[18] Gruswitz F., O'Connell J., Stroud R.M. (2007). Inhibitory complex of the transmembrane ammonia channel, AmtB, and the cytosolic regulatory protein, GlnK, at 1.96 A. Proc Natl Acad Sci U S A.

[19] Bandyopadhyay A., Arora A., Jain S., Laskar A., Mandal C., Ivanisenko V.A., Fomin E.S., Pintus S.S., Kolchanov N.A., Maiti S., Ramachandran S. (2010). Expression and molecular characterization of the *Mycobacterium tuberculosis* PII protein. J Biochem.

[20] Atkinson M.R., Kamberov E.S., Weiss R.L., Ninfa A.J. (1994). Reversible uridylylation of the *Escherichia coli* PII signal transduction protein regulates its ability to stimulate the dephosphorylation of the transcription factor nitrogen regulator I (NRI or NtrC). J Biol Chem.

[21] Beckers G., Strosser J., Hildebrandt U., Kalinowski J., Farwick M., Kramer R., Burkovski A. (2005). Regulation of AmtR-controlled gene expression in *Corynebacterium glutamicum*: mechanism and characterization of the AmtR regulon. Mol Microbiol.

[22] Nolden L., Ngouoto-Nkili C.E., Bendt A.K., Kramer R., Burkovski A. (2001). Sensing nitrogen limitation in *Corynebacterium glutamicum*: the role of *glnK* and glnD. Mol Microbiol.

[23] Tiffert Y., Franz-Wachtel M., Fladerer C., Nordheim A., Reuther J., Wohlleben W., Mast Y. (2011). Proteomic analysis of the GlnR-mediated response to nitrogen limitation in *Streptomyces coelicolor* M145. Appl Microbiol Biotechnol.

[24] Amon J., Brau T., Grimrath A., Hanssler E., Hasselt K., Holler M., Jessberger N., Ott L., Szokol J., Titgemeyer F., Burkovski A. (2008). Nitrogen control in *Mycobacterium smegmatis*: nitrogen-dependent expression of ammonium transport and assimilation proteins depends on the OmpR-type regulator GlnR. J Bacteriol.

[25] Jenkins V.A., Robertson B.D., Williams K.J. (2012). Aspartate D48 is essential for the GlnR-mediated transcriptional response to nitrogen limitation in *Mycobacterium smegmatis*. FEMS Microbiol Lett.

[26] Carroll P., Pashley C.A., Parish T. (2008). Functional analysis of GlnE, an essential adenylyl transferase in *Mycobacterium tuberculosis*. J Bacteriol.

[27] Parish T., Stoker N.G. (2000). glnE is an essential gene in *Mycobacterium tuberculosis*. J Bacteriol.

[28] Harper C.J., Hayward D., Kidd M., Wiid I., van Helden P. (2010). Glutamate dehydrogenase and glutamine synthetase are regulated in response to nitrogen availability in *Mycobacterium smegmatis*. BMC Microbiol.

[29] Harth G., Maslesa-Galic S., Tullius M.V., Horwitz M.A. (2005). All four *Mycobacterium tuberculosis glnA* genes encode glutamine synthetase activities but only GlnA1 is abundantly expressed and essential for bacterial homeostasis. Mol Microbiol.

[30] Rengarajan J., Bloom B.R., Rubin E.J. (2005). Genome-wide requirements for *Mycobacterium tuberculosis* adaptation and survival in macrophages. Proc Natl Acad Sci U S A.

[31] Parish T.S., Neil G. (1998). Mycobacterial protocols.

[32] Read R., Pashley C.A., Smith D., Parish T. (2007). The role of GlnD in ammonia assimilation in *Mycobacterium tuberculosis*. Tuberculosis.

[33] Livak K.J., Schmittgen T.D. (2001). Analysis of relative gene expression data using real-time quantitative PCR and the 2(−Delta Delta C(T)) method. Methods.

[34] Smyth G.K. (2004). Linear models and empirical Bayes methods for assessing differential expression in microarray experiments. Stat Appl Genet Mol Biol.

[35] Li Y., Al-Eryani R., Yarbrough M.L., Orth K., Ball H.L. (2011). Characterization of AMPylation on threonine, serine, and tyrosine using mass spectrometry. J Am Soc Mass Spectrom.

[36] Domon B., Aebersold R. (2006). Mass spectrometry and protein analysis. Science.

[37] Fink D., Falke D., Wohlleben W., Engels A. (1999). Nitrogen metabolism in *Streptomyces coelicolor* A3(2): modification of glutamine synthetase I by an adenylyltransferase. Microbiology.

[38] Amon J., Titgemeyer F., Burkovski A. (2009). A genomic view on nitrogen metabolism and nitrogen control in mycobacteria. J Mol Microbiol Biotechnol.

[39] Burkovski A. (2003). Ammonium assimilation and nitrogen control in *Corynebacterium glutamicum* and its relatives: an example for new regulatory mechanisms in actinomycetes. FEMS Microbiol Rev.

[40] Fisher S.H. (1999). Regulation of nitrogen metabolism in *Bacillus subtilis*: vive la difference!. Mol Microbiol.

[41] Jiang P., Ninfa A.J. (2009). Alpha-ketoglutarate controls the ability of the *Escherichia coli* PII signal transduction protein to regulate the activities of NRII (NrB but does not control the binding of PII to NRII). Biochemistry.

[42] Behrends V., Williams K.J., Jenkins V.A., Robertson B.D., Bundy J.G. (2012). Free glucosylglycerate is a novel marker of nitrogen stress in *Mycobacterium smegmatis*. J Proteome Res.

[43] Bueno R., Pahel G., Magasanik B. (1985). Role of *glnB* and *glnD* gene products in regulation of the *glnALG* operon of *Escherichia coli*. J Bacteriol.

[44] Pioszak A.A., Jiang P., Ninfa A.J. (2000). The *Escherichia coli* PII signal transduction protein regulates the activities of the two-component system transmitter protein NRII by direct interaction with the kinase domain of the transmitter module. Biochemistry.

[45] Tiffert Y., Supra P., Wurm R., Wohlleben W., Wagner R., Reuther J. (2008). The *Streptomyces coelicolor* GlnR regulon: identification of new GlnR targets and evidence for a central role of GlnR in nitrogen metabolism in actinomycetes. Mol Microbiol.

[46] Merrick M.J., Edwards R.A. (1995). Nitrogen control in bacteria. Microbiol Rev.

[47] Rehm N., Buchinger S., Strosser J., Dotzauer A., Walter B., Hans S., Bathe B., Schomburg D., Kramer R., Burkovski A. (2010). Impact of adenylyltransferase GlnE on nitrogen starvation response in *Corynebacterium glutamicum*. J Biotechnol.

[48] Atkinson M.R., Ninfa A.J. (1998). Role of the GlnK signal transduction protein in the regulation of nitrogen assimilation in *Escherichia coli*. Mol Microbiol.

[49] Mehta R., Pearson J.T., Mahajan S., Nath A., Hickey M.J., Sherman D.R., Atkins W.M. (2004). Adenylylation and catalytic properties of *Mycobacterium tuberculosis* glutamine synthetase expressed in *Escherichia coli* versus mycobacteria. J Biol Chem.

