# Supplementary figures and images for: Adenylylation of mycobacterial Glnk (PII) protein is induced by nitrogen limitation
Source: Tuberculosis (Edinb). 2013 Mar;93(2):198–206. doi: 10.1016/j.tube.2012.12.003 (PMC3612183; doi:10.1016/j.tube.2012.12.003)

## Slide 1
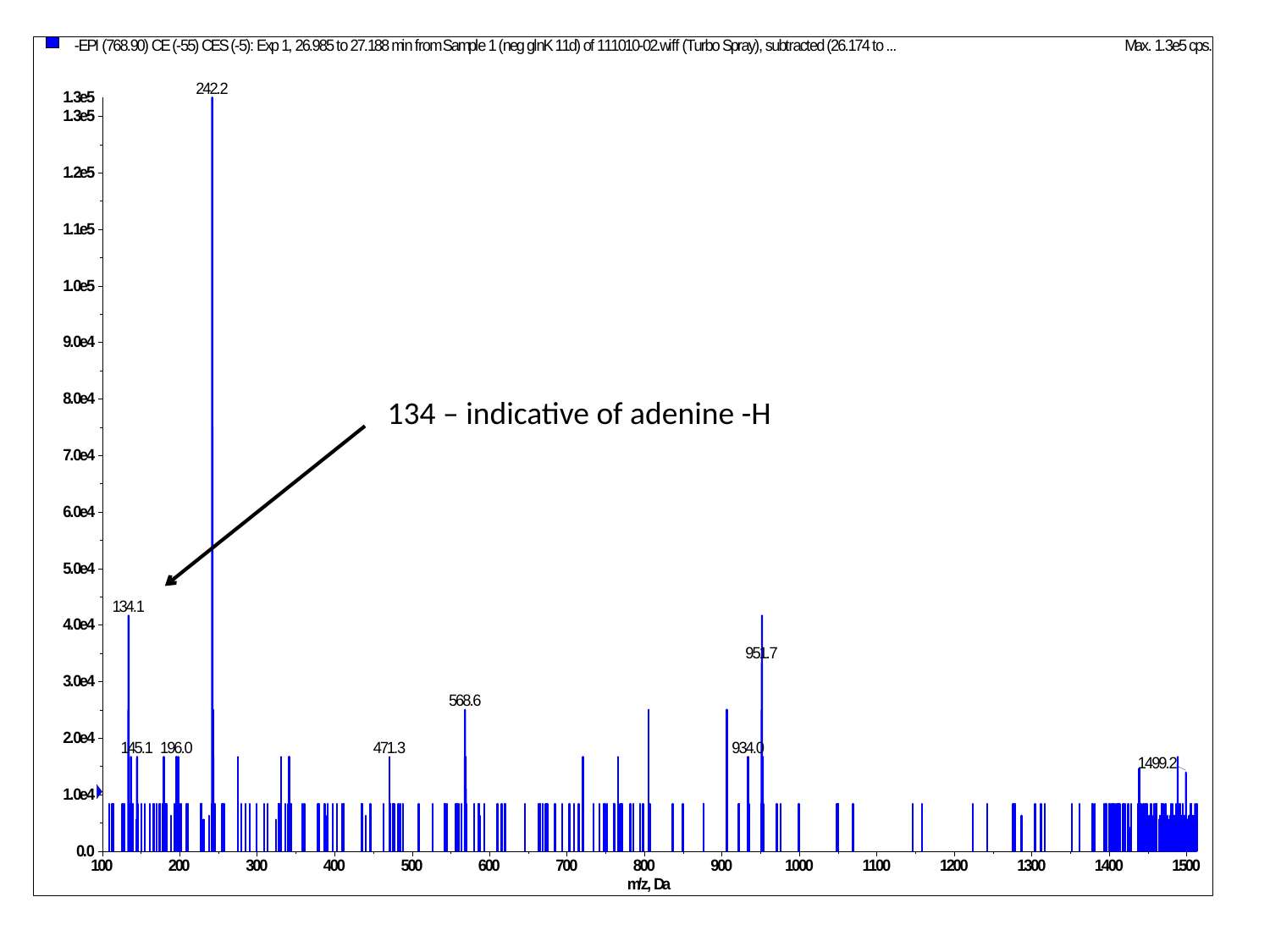

134 – indicative of adenine -H

Supplement: Figure S1 — Negative ionisation MS spectra of the GlnK peptide obtained from M. smegmatis grown in nitrogen limitation showing a strong product ion of 134 indicative of adenylylation. [file mmc2.pptx]

## Slide 1
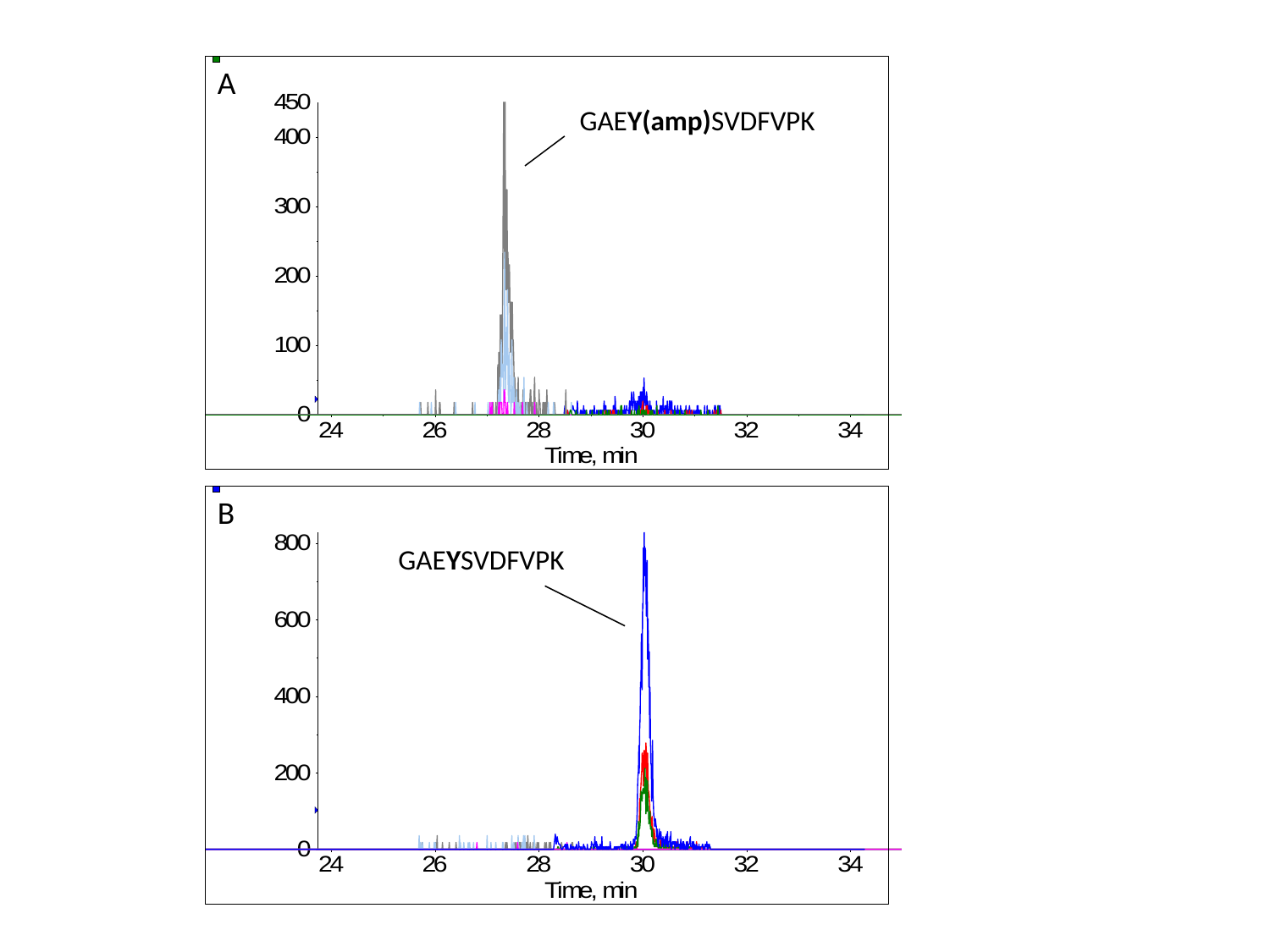

A
GAEY(amp)SVDFVPK
B
GAEYSVDFVPK

Supplement: Figure S2 — MRM analysis of the GlnK peptide obtained from M. tuberculosis grown in nitrogen limitation A. Wild type strain B.glnD deletion strain. [file mmc3.pptx]

## Slide 1
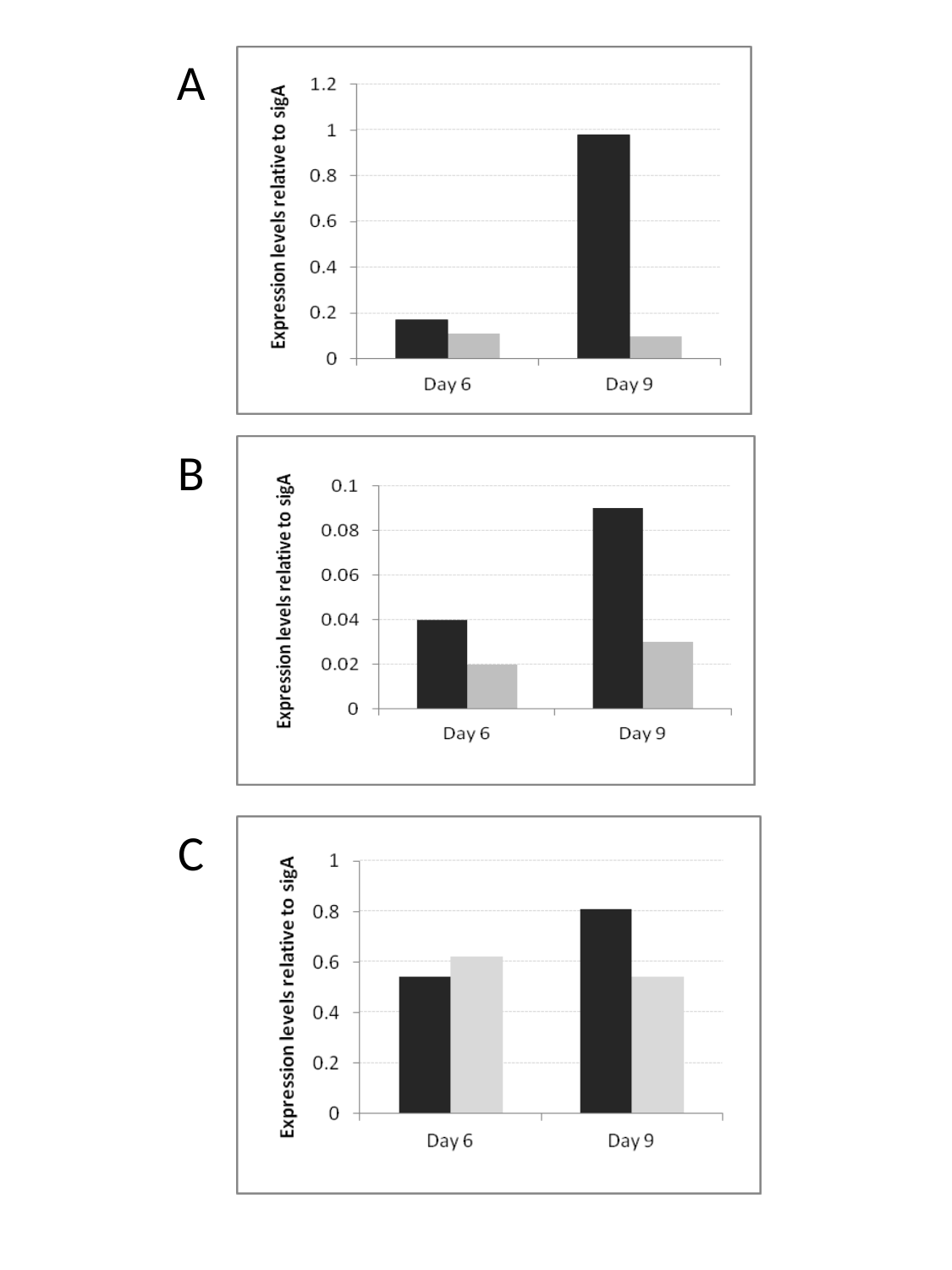

A
B
C

Supplement: Figure S3 — Confirmation by qRT-PCR of the induction of nitrogen response genes (A) nirB (B) amtB and (C) glnK in the wild type strain grown in nitrogen limitation for nine days. Data is a representative result of three experiments. Low (1 mM) ammonium black bars, High (30 mM) ammonium grey bars. [file mmc4.pptx]
