# Supplementary material for: Adenylylation of mycobacterial Glnk (PII) protein is induced by nitrogen limitation
Source: Tuberculosis (Edinb). 2013 Mar;93(2):198–206. doi: 10.1016/j.tube.2012.12.003 (PMC3612183; doi:10.1016/j.tube.2012.12.003)
Supplement: Supplementary file 5 [file mmc5.docx]

**Table S1.** Significantly induced genes (fold change >2 and p value < 0.01) in the *M. tuberculosis* *glnD* deletion strain compared to wild type in nitrogen limitation.

| **Gene ID** | **Gene Name** | **Gene Function** | **p value** | **Fold change**  **(*glnD* ko vs WT)** |
| --- | --- | --- | --- | --- |
| Rv2386c | *mbtI* | isochorismate synthase | 0.003 | 3.249 |
| Rv2382c | *mbtC* | polyketide synthetase | 0.001 | 2.805 |
| Rv1755c | *plcD* | phospholipase C 4 | 0.004 | 2.673 |
| Rv1552 | *frdA* | fumarate reductase flavoprotein subunit | 0.006 | 2.507 |
| Rv1555 | *frdD* | fumarate reductase membrane anchor subunit | 0.008 | 2.226 |
| Rv3402c | *-* | conserved hypothetical protein | 0.005 | 2.177 |
| Rv1519 | *-* | conserved hypothetical protein | 2.51 x 10^-5^ | 2.174 |
| Rv2251 | *-* | flavoprotein | 0.008 | 2.070 |
| Rv2383c | *mbtB* | phenyloxazoline synthase | 0.004 | 2.054 |

**Table S2.** Significantly repressed genes (fold change <0.5 and p value < 0.01) in the *M. tuberculosis* *glnD* deletion mutant compared to wild type in nitrogen limitation.

| **Gene ID** | **Gene Name** | **Gene Function** | **p value** | **Fold change**  **(*glnD* ko vs WT)** |
| --- | --- | --- | --- | --- |
| Rv1120c | - | conserved hypothetical protein | 0.002 | 0.070 |
| Rv1779c | - | hypothetical membrane protein | 0.004 | 0.193 |
| Rv3841 | *bfrB* | bacterioferritin | 1.89 x 10^-5^ | 0.223 |
| Rv2428 | *ahpC* | alkyl hydroperoxide reductase C protein | 0.002 | 0.226 |
| Rv1854c | *ndh* | NADH dehydrogenase | 0.002 | 0.229 |
| Rv2429 | *ahpD* | alkyl hydroperoxide reductase D protein | 0.006 | 0.318 |
| Rv3140 | *fadE23* | acyl-CoA dehydrogenase | 0.008 | 0.368 |
| Rv2940c | *mas* | multifunctional mycocerosic acid synthase | 0.002 | 0.390 |
| Rv2107 | *PE22* | PE family protein | 0.003 | 0.410 |
| Rv0146 | *-* | conserved hypothetical protein | 0.001 | 0.439 |
| Rv1057 | *-* | conserved hypothetical protein | 0.004 | 0.457 |
| Rv3484 | *cpsA* | hypothetical protein | 0.006 | 0.463 |
| Rv0483 | *lprQ* | lipoprotein | 0.006 | 0.465 |
| **Rv2918c** | ***glnD*** | **adenyl transferase** | **4.40 x 10^-6^** | **0.087^a^** |

**^a^** Probe BUGS0000000577399 was removed from the analysis due to transcription run through from the upstream gene. Deletion of *glnD* in the mutant was also confirmed by qRT-PCR (data not shown).

**Table S3.** Custom Taqman *M. tuberculosis* gene expression primer and probe sequences used in this study.

| **Gene** | **Forward primer (5’-3’)** | **Reverse primer (5’-3’)** | **FAM Reporter probe (5’-3’)** |
| --- | --- | --- | --- |
| *amtB* (Rv2920) | CTGTTTCGACCGCACAATCTG | CCGTACCAGCCGAACCA | CCGAGCATCACAAACG |
| *nirB* (Rv0252) | CCGGATTCGGCCCAGAT | CTTCAGCTCGCCCTTGGT | CTGCTCGTGCAACAAC |
| *glnK* (Rv2919) | GGCCACACGGAGGTTTACC | AATCGTCAACAACGACCTCGAT | TCGGTACGAAATCCAC |
| *glnA1* (Rv2220) | GACGACATGCAGTTGTACAAGTAC | GTGACCGTTTTGCCGTTCTG | CAGGCGGTGTTCTTG |
| *sigA* (Rv2703) | GCCACGCAGCTGATGAC | GCGGCAGGCAGCTTT | TCGCCGCGCTCGCTAA |
| *glnD* (Rv2918) | CCGAACCGGGCTCTGG | GCGGAAGGTCGCGAATG | ATTCCGGCAACAACC |
